# Supplementary material for: Are carbon emissions trading and green financial instruments synergistic? -Comprehensive quantitative research based on content analysis
Source: PLoS One. 2024 Mar 7;19(3):e0298601. doi: 10.1371/journal.pone.0298601 (PMC10919601; doi:10.1371/journal.pone.0298601)
Supplement: S1 Data — (ZIP) [file pone.0298601.s001.zip › Data/地级市Z_F工作报告.zip!/░▓╗╒/░÷▓║╩╨/└┤╫╘╣1⁄2╓┌║┼í╛┬φ┐╦╩2╛▌═°í┐_2017.docx]

各位代表： 

　　现在，我代表市人民政府向大会报告工作，请予审议，并请市政协委员和其他列席人员提出意见。 

　　一、克难攻坚，奋力实现“十三五”良好开局 

　　2016年，面对错综复杂的国内外形势，我们认真学习贯彻习近平总书记系列重要讲话特别是视察安徽重要讲话精神，在中共蚌埠市委的坚强领导下，开拓创新，锐意进取，较好地完成了市十五届人大四次会议确定的目标任务。 

　　——经济运行稳中向好。11项主要经济指标中，6项总量、9项增速位居全省前5位。全市地区生产总值1385.8亿元，同比增长9.4%、居全省第3位，连续22个季度高于全省平均水平。财政收入251.2亿元，增长10%。规上工业企业1162户，实现增加值743.2亿元、居全省第3位，增长9.9%、居全省第4位。粮食产量280.5万吨，实现“十三连丰”。蝉联“全国电商百佳城市”。 

　　——“两个中心”定位确立。省委、省政府提出“支持蚌埠建成淮河流域和皖北地区中心城市”，淮河生态经济带建设纳入国家和省“十三五”规划，蚌埠发展定位更高、空间更大、机遇更多。 

　　——科技创新取得突破。成功纳入国家系统推进全面创新改革试验，合芜蚌国家自主创新示范区启动建设。荣获全省唯一中国产学研合作促进奖，获批全省首个国家知识产权质押融资示范城市。世界最薄0.15毫米信息显示触控玻璃实现量产，蚌埠玻璃工业设计研究院第三次获国家科技进步二等奖，彭寿获杰出工程师奖。大富光电OLED显示器及组件列入省重大新兴产业专项。战略性新兴产业产值突破千亿，占规上工业33.2%、居全省第1位。 

　　——深化改革亮点频现。“一户一块田”试点、公共资源交易“八统一”改革受到肯定，在全国推介。荣获国家“公立医院综合改革真抓实干成效明显予以激励支持城市”。入选全国首批商务综合行政执法体制改革试点市。 

　　——脱贫攻坚首战告捷。88个贫困村全部出列，39778名贫困人口脱贫，超额完成年度目标任务。 

　　——人民生活持续改善。城镇、农村常住居民人均可支配收入分别增长8.7%、9%，居全省第4位、第1位。新增城镇就业8.32万人，占全省1/8。城乡低保全面提标，企业退休职工养老金“十二连增”。完成48个省、市重点计划老旧小区整治提升和12个集贸市场标准化改造。 

　　一年来，主要做了以下工作： 

　　（一）综合施策稳增长，经济保持较快发展。全面落实“营改增”等国家结构性减税和普遍性降费政策，减免抵退各项税费50亿元。兑现援企稳岗帮扶资金6639万元。“4321”政银担在保余额30.44亿元，新增13.71亿元。续贷转贷过桥资金、“税融通”分别放款27.93亿元、6.21亿元。本外币贷款余额1406.2亿元，增长17.2%。组建50亿元“淮河兴业”退市进园融资平台，首期30亿元硅基新材料产业发展基金投入运营，设立安徽高新赛伯乐电子信息、育珠设计等9个产业投资引导基金，规模达130.85亿元。争取省代发地方政府债券64.6亿元。新增新三板挂牌企业9家，双环电子创业板上市获受理。固定资产投资1666亿元，增长14.3%。北方通用MEMS产业化基地一期、新知科技产业园一期等125个重点项目建成，中光电8.5代TFT-LCD玻璃基板、泰富重装华东智能制造产业园等217个重点项目开工建设。 

　　（二）创新驱动调结构，产业升级步伐加快。一、二、三产增加值分别增长3.7%、9.3%、11.5%，二三产业占地区生产总值比重提高0.6个百分点。省级硅基新材料产业集聚发展基地综合考核居全省第4位。市级生物制造、智能装备制造、专用电子器件基地启动建设。新认定国家高新技术企业37家，高新技术产业增加值310.8亿元、增长24%。新组建省级以上研发平台15家，新增院士工作站5家，与中科院合作设立科技成果交易转化孵化中心。全社会研发经费投入27.28亿元，增长16.5%。万人有效发明专利拥有量8.6件，增长34.5%。高新闪创空间、蓝莓星创天地纳入国家级众创空间，青年创业园入围全国创业孵化示范基地。5家西部化工企业退市进园协议全部签订，丰原集团、中粮生化、天润化工入园项目相继开工，丰原集团年产1万吨明胶和环酸项目竣工投产。蚌埠铜陵（五河）现代产业园获省批复。社会消费品零售总额644亿元，增长12.9%。银泰城、海吉星二期等投入运营。旅游总收入197亿元，增长21.4%。新增怀远香飘飘、五河新一禾、固镇百万只肉羊等一二三产融合项目。200亩以上土地流转面积占总流转面积44%。新增国家级农业合作示范社3家，省级示范社6家、示范家庭农场14家。连续18年实现耕地占补平衡。五河县荣获全国节约集约用地模范县称号。 

　　（三）改革开放添活力，发展环境持续优化。深化商事制度改革，全面推进“五证合一、一照一码”，新增注册企业7644户、认缴资本565亿元，分别增长25%、24%。全面完成不动产登记制度改革。农村土地承包经营权确权登记颁证、国有林场改革基本完成。“一户一块田”试点6.87万亩。市县区全面实行公共资源交易“八统一”。专利权质押贷款突破8亿元。省级“多规合一”试点基本完成。加强与央企合作，中建材太阳能薄膜电池用高温玻璃项目落地开工。新签约高桥特种防火电缆等知名民企合作项目81个，项目数、投资额均居全省第3位。比亚迪、武汉光谷蓝焰等知名企业落户蚌埠。实际到位市外亿元以上项目资金820亿元、增长10.1%；实际利用外商直接投资15亿美元，增长8%。实现进出口总额17.6亿美元。蚌埠港二类水运口岸获批筹建。保税物流中心业务量超2亿美元、增长45%。外事、侨务、对台等工作促进了对外招商引资。 

　　（四）统筹城乡促协调，人居环境不断改善。实施大建设项目147个，完成投资256亿元。新启动棚改项目40个，回迁安置2.28万户。开工建设各类保障性安居工程24086套，分配入住9026套。长淮卫淮河大桥和G104五河段、S306沫河口段、S307怀远段等连接通道改造竣工通车。二钢新村、大禹家园等“海绵城市”和凤阳东路、治淮东路等地下综合管廊试点项目开工建设。2015年度创建全国文明城市测评成绩位居前列。发挥工会、共青团、妇联、文联、老龄委、关工委等群团和社会组织作用，广泛开展“诚信蚌埠·孝善珠城”、“我爱我家·行走蚌埠”等活动。完成4条商业大街、12个重点乡镇整体提升改造。开展“毁绿种菜”专项整治，恢复绿地2.9万m2。新增路边停车泊位3965个。数字化城管系统正式启用。实施大气污染防治攻坚行动，大力开展工业废气、城市扬尘、机动车尾气等综合治理。在全省率先建立水污染跨行政区域联防联控体系。“十二五”最严格水资源管理考核居全省第1位。三汊河湿地获批国家级湿地公园。津浦大塘公园等161个园林绿化提升项目完成。新增绿地344.9万m2，改造绿地319.3万m2。成片造林7.5万亩，补植补造5.83万亩。美国白蛾防治取得明显成效。启动27个乡镇政府驻地建成区整治建设，20个美丽乡村省级中心村建设项目全面完成。固镇县、五河县荣获全省美丽乡村建设工作先进县，我市综合成绩居全省第3位。 

　　（五）倾心服务惠民生，社会大局和谐稳定。投入资金55.2亿元，完成33项民生工程。发放残疾人救助资金5341万元。补助“一户多残”家庭445万元。城乡居民医保补助标准提高至420元。城市、市区农村、三县农村低保标准年分别提高720元、1020元、780元。住房公积金缴存率达63%，居全省第2位。新建、改扩建幼儿园49所、中小学校183所。资助各类贫困学生31261人、资金5270万元。市一院综合病房大楼辅楼和市三院、蚌医一附院全科医生临床培训基地基本建成。现代泗州戏《绿皮火车》荣获第五届全国少数民族文艺会演剧目银奖。大型原创民族舞剧《大禹》在安徽大剧院精彩上演。美术馆、少儿图书馆建成开放。市体育中心、游泳跳水馆等场馆开工建设，成功举办第十四届市运会暨首届全民健身运动会。统计、气象、档案、地方志、社会科学等服务经济社会发展能力进一步增强。民族宗教工作继续加强。全民国防教育、国防后备力量建设扎实推进，实现全国双拥模范城“六连冠”。深入推进安全生产“铸安”行动，各类事故起数、死亡总人数、较大事故起数分别下降14.2%、18.5%、33.3%。对全市1047家餐饮单位进行油烟设施改造，打造龙子湖区延安北路、经开区蓝天路等8个小餐饮示范街（区），荣获省首批食品药品安全城市。防空、防震、消防、应急等减灾防灾体系更加健全。特警张劼荣膺全国“公安楷模”、“中国好人”。启动全国和谐社区建设示范城市创建活动。化解信访积案929件，上访总量下降28.6%。社会治安视频监控系统实现主城区重点区域全覆盖。严厉打击各类违法犯罪，人民群众安全感持续提升。 

　　各位代表！ 

　　过去的一年，我们付出的努力异常艰辛，收获的成果弥足珍贵。这是党中央、国务院，省委、省政府和市委正确领导的结果，是全市人民团结奋斗的结果。我代表市人民政府，向全市人民，向人大代表、政协委员，向离退休老同志，向各民主党派、工商联、无党派人士，向各人民团体，向驻蚌解放军指战员、武警官兵和政法公安干警，向上级部门和驻蚌单位，向所有关心和支持蚌埠改革发展的各界朋友，表示崇高的敬意和衷心的感谢！ 

　　我们也清醒地看到，我市经济社会发展中还存在不少困难和问题。创新发展牵动力不强，科教资源、研发成果还没有充分转化为产业优势，激励创新创业的政策环境有待优化。工业经济主导地位不突出，龙头企业少，大项目少，知名品牌少。部分工业园区主导产业不明晰，基础设施特别是环保设施建设滞后。重大项目储备偏小、偏少，投资后劲不足。债务、金融等领域特别是非法集资、电信网络诈骗的风险隐患依然存在。现代服务业发展不快，电商、健康养老等新业态、新模式发展不充分。通达辐射能力和集聚虹吸效应有限，教育、医疗等基本公共服务供给总量不足与质量不优并存，中心城市能级亟需提升。生产、消防、食品领域安全隐患仍然较多，环境污染、交通拥堵治理任务艰巨。县域经济综合实力不强、总量不大，农业产业化特别是农产品精深加工短板突出。脱贫攻坚任务较为繁重，等等。针对这些问题，我们将自我加压，采取有力措施，认真加以解决，决不辜负人民的期待！ 

　　二、聚力担当，深入实施五大发展行动计划 

　　当前，世界经济进入发展转型期，我国经济发展新常态特征更加明显。国家深入实施“三大战略”，全面发力供给侧结构性改革，系统推进全面创新改革试验，加快建设合芜蚌国家自主创新示范区，加速发展中原经济区、淮河生态经济带等，一系列优势和机遇在蚌埠交汇叠加。经过多年来特别是“十二五”时期的发展，蚌埠创新发展有优势、协调发展有后劲、绿色发展有潜力、开放发展有机遇、共享发展有基础。从现在起到2020年，是蚌埠建成“两个中心”的关键时期、实现“两个全面”的决胜阶段，我们将牢记使命担当，坚定不移用新发展理念统领全局，深入实施五大发展行动计划，立足自身资源优势，以解决突出问题为突破口和主抓手，抢抓机遇，加快发展，把长板拉得更长，把短板补齐补强，加快建设经济强、城市美、百姓富、环境优、文明程度高的新蚌埠。 

　　一是实施创新发展行动，在创新驱动发展上走在前列。高举创新大旗，全力推动合芜蚌国家自主创新示范区建设，系统推进全面创新改革试验，加快建设具有重要影响力的区域产业创新中心和科技成果交易转化中心，争创国家级创新型城市。着力创建国家级硅基新材料和生物产业创新中心，推动合芜蚌金融服务自主创新综合改革、人才管理改革试验区建设，统筹建设科教强市、技工强市、制造强市。市高新区、蚌埠工业园区建成千亿级园区，蚌埠经济开发区、怀远经济开发区争创国家级开发区。统筹推进“三重一创”，培育4个千亿级战略性新兴产业集群。提升现代服务业水平，加快发展现代生态农业。二三产业占地区生产总值比重超过90%。 

　　二是实施协调发展行动，在中心城市能级提升上干在实处。巩固和放大区域中心、交通枢纽、工业大市、商贸重镇、科教之城、创新高地优势，着力推进基础设施再完善、征迁棚改全扫尾、公共服务提质效、城乡面貌增颜值，持续提升中心城市集聚和虹吸效应。城镇化率提高到60%以上，基本公共服务实现常住人口全覆盖。积极融入合肥都市圈、南京都市圈，推动合淮蚌城市带建设。配套完善主城区功能，推动城南、高铁、淮上、滨湖、马城等组团发展。围绕“皖北领先、省内进位”要求，大力实施县域经济振兴工程。依托国家级大禹文化产业示范园区，加快建设特色文化名城。提高城市文明程度和市民综合素质，全面建设全国文明城市。 

　　三是实施绿色发展行动，在生态文明建设上打造样板。把建设人水和谐、绿色共享的淮河生态经济带作为绿色发展的引领工程，扎实推进国家级生态文明先行示范区建设，创建国家水生态文明城市、国家节水型城市和国土资源节约集约模范市。做活水文章，全面实施淮河蚌埠段主要干支流污染治理、方邱湖堤防退建和主要河湖水系连通工程，推进怀洪新河水系洼地治理、大型灌区建设，打造三汊河国家湿地公园和沱湖湿地自然保护区绿色发展名片，构成“河畅、水清、岸绿、景美”的水生态体系。创建安徽省森林城市，森林覆盖率达20%以上，建成区绿化覆盖率达44%。 

　　四是实施开放发展行动，在双向开放合作上闯出新路。积极融入国家“三大战略”，坚持“引进来”、“走出去”，加快大通道、大平台、大通关建设，把蚌埠打造成为皖北对外开放门户、淮河生态经济带对外开放的重要战略支撑、京沪线上对外开放的重要节点城市。统筹实施民用机场、铁路、高速公路、航道提升等重点工程，加快构建立体交通体系和零换乘系统。积极探索与沪苏浙合作共建开发园区。提升保税物流中心、铁路无水港运营水平，建设皖北首家综合保税区，积极参与省争创自贸区。推进国家级电子商务与物流快递协同发展试点，建设跨境电商产业园，建成区域性商贸物流中心。 

　　五是实施共享发展行动，在小康路上一个都不能掉队。全面完成脱贫攻坚任务，现行标准下农村贫困人口全部脱贫，怀远县摘帽。就业、健康、养老、社保、住房等保障体系更加健全，教育、医疗、文化等公共服务水平稳步提高。深入实施全民健身计划，全力承办好第十四届省运会，打造群众体育大市、竞技体育强市。支持分享经济发展，提高资源利用效率。打造军民融合产业发展基地和龙头企业，推进军民深度融合发展。全力推动公共安全、生产安全、食品药品安全建设，让人民群众物质更富足、精神更富有、生活更幸福！ 

　　三、加压奋进，全力做好2017年重点工作 

　　今年政府工作的总体要求是：全面贯彻党的十八大和十八届三中、四中、五中、六中全会精神，深入贯彻落实习近平总书记系列重要讲话特别是视察安徽重要讲话精神，坚持稳中求进工作总基调，围绕建成“两个中心”、实现“两个全面”目标，以落实新发展理念为主题，以提高发展质量和效益为中心，以深化供给侧结构性改革为主线，以实施五大发展行动计划为主抓手，始终以“蹦起来摘桃子”的精气神，全面做好稳增长、促改革、调结构、惠民生、防风险各项工作，保持经济中高速增长、产业迈向中高端水平、社会大局和谐稳定。 

　　经济社会发展的主要预期目标是：地区生产总值增长9.5%左右；一、二、三产分别增长3.5%以上、10%以上和11%，规上工业增加值增长10.5%；财政收入增长9.5%；固定资产投资增长14%以上；全社会研究与试验发展经费支出增长16.5%，万人有效发明专利拥有量超过10件；社会消费品零售总额增长12.5%；进出口总额增长高于全省平均水平；城镇常住居民人均可支配收入增长与经济增长同步，农村常住居民人均可支配收入增长10%；城镇登记失业率、居民消费价格涨幅、单位生产总值能耗、国家要求削减的主要污染物排放均控制在省下达的目标以内。 

　　（一）深入实施创新驱动发展战略，激发经济增长内生动力 

　　做强创新平台。紧抓领军企业、重点项目、产业链条、产业集聚四大环节，依托蚌埠玻璃工业设计研究院、中国电科40、41所、兵器工业集团214所、丰原集团发酵技术国家工程技术研究中心、中粮生化技术中心等科研院所，实施“0.15mm以下极薄浮法电子玻璃工艺及产业化关键技术”、“航空装备用大功率微波开关”、“智能多传感数据融合MPSoC处理芯片”、“生物新材料”、“微生物菌剂”等科技重大专项。加强面向企业的研发机构建设，规上工业企业设立研发机构比例达35%，科技企业孵化器面积达40万m2，新认定高新技术企业25家以上。创建省级硅基新材料制造业创新中心，筹建江淮智能汽车关键技术研究中心、甲骨文科技创新园产学研基地，新建省级以上研发平台10个。 

　　加速成果转化。推进中科院科技成果交易转化孵化中心、绿色谷技术创新中心建设，建成创新一站式云服务大厅，争创科技创新走廊。安排1亿元专项资金，支持驻蚌高校和科研院所等引进国内外高层次人才、加大科技研发投入、孵化科技成果，建设一批综合性高水平的科技创新基地。建立与驻蚌高校院所部队联席会议制度，研究部署项目对接、建设等重大事项，打通科技成果转化“关键一公里”。新增高新技术产品120件，技术合同交易额突破20亿元。 

　　强化创新合作。完善需求衔接和管理机制，在军民融合产业发展、基础设施共建共用、科技协同创新、联合应急救援等方面拓展广度和深度，争创国家军民深度融合创新示范区。推进双环电子、方圆机电等 “民参军、军转民”项目产业化进程。建立大型仪器（设备）共享平台和信息技术交流平台。建设高端人才公寓、学术交流中心。依托后青春工业设计研究院创建国家级工业设计平台，打造工业设计、硅基新材料等一批有特色、高水平的学术交流论坛。 

　　营造创新环境。推进金融与科技融合，合肥科技农商行蚌埠分行开业运营。制定重大装备首台（套）突破及示范应用补助、小微企业科技创新券等政策。加强知识产权保护，加大侵权打击力度。大力实施人才新政，推进科技成果使用权、处置权和收益权“三权”改革，落实科技成果转化收益不低于70%用于奖励重要贡献人员和团队等政策，加快合芜蚌人才金港、蚌埠人才特区建设。完善人才评价机制，推进职称评审社会化、专业化分类改革。

　　（二）着力发展实体经济，加快建设工业强市 

　　培育壮大战略性新兴产业。加快构建 “1+3”创新产业体系，硅基新材料、生物制造、智能装备制造、专用电子器件产业基地产值分别突破500亿元、400亿元、400亿元、600亿元。紧盯尖端领域、前沿技术，实施大富光电OLED显示器及组件等重大专项，研究建立“1+3”创新产业研究院，精准强化源头创新。开工建设乐朋年产100万吨安瓿聚酯瓶片、华特科技10KK/月指纹识别模组等项目，加快推进中光电8.5代TFT-LCD玻璃基板、中国电科41所现代通信仪器及自动化装备制造中心、泰富重装华东智能制造产业园、鸿路钢构产业园和丰原集团年产30万吨氨基酸、15万吨聚乳酸、12万吨维生素、20万吨有机酸等项目，建成比亚迪云轨安徽制造中心、中建材光电触摸屏用保护玻璃、凯盛光伏铜铟镓硒薄膜太阳能电池一期、兵器工业集团214所EMCCD器件等项目。战略性新兴产业产值达1200亿元，占规上工业产值比重达34%。 

　　大力改造提升传统产业。开展“互联网+制造”行动，推动新一代信息技术与制造技术融合发展。改造升级智能工厂或数字化车间10个。新增专精特新企业40户、两化融合示范企业30户。鼓励企业运用零地技改、机器换人等模式，促进传统产业改造升级和产能提升。建成国电蚌埠电厂二期、蚌埠卷烟厂易地技改项目。加快推进西部化工企业退市进园，建设八一化工、佳先股份搬迁项目，续建天润化工聚丙烯酰胺等项目，竣工投产中粮生化燃料乙醇及供热、丰原集团热电联产一期项目。支持沫河口工业园培育高端医药产业。 

　　全力扶持实体经济发展。围绕大型骨干企业和专精特新企业发展、生产经营困难企业帮扶、规模以下企业成长，常态化开展“行走企业”活动，建立“千干扶千企”服务机制，分类走访调研，实行清单管理，逐一销号解决问题。实施打造千亿产业千亿园区、培育百亿企业百年老店计划。蚌埠玻璃工业设计研究院、中粮生化实现百亿突破。净增规上工业企业200户。支持安徽水利、中城建六局2家百亿建筑企业发展壮大，净增资质等级以上建筑企业15户。开展质量品牌示范年活动，推进企业增品种、提品质、创品牌行动，新培育省级以上工业精品、新产品20个。开展首届“蚌埠知名品牌”评审，新增省级以上著名商标20件、名牌产品10个。建设滤清器等国家质检中心。 

　　（三）深化供给侧结构性改革，促进经济持续健康较快发展 

　　深入推进“去降补”。开展严禁新增过剩产能专项整治，严禁违规审批、建设限制类和淘汰类项目。建立健全“僵尸企业”数据库，实现50%以上“僵尸企业”出清重组。推进市区城中村改造全面货币化，棚改货币化安置比例70%以上。完成去化商品房库存70万m2。引导符合规划要求的商业办公用房转为科技孵化器、众创空间、养老和服务设施等。推进“4321”政银担风险分担机制，设立融资担保风险补偿专项资金。鼓励各银行业金融机构开展基于风险评估的“循环贷”，续贷转贷过桥资金、“税融通”业务规模达20亿元、6亿元。新增申报上市企业1家、新三板挂牌企业10家，证券化率提高5个百分点。新增贷款210亿元、直接融资100亿元。全面抓好省降成本20条、市降成本35条政策落实，降低企业用能、用地、物流、人工等成本。围绕产业发展、公共服务、民生保障等方面的薄弱环节，推进补短板项目建设。 

　　加大有效投资力度。坚决破除制约民间投资体制机制障碍，放宽非公有制经济市场准入，抓好支持民营经济发展的各项政策落实。扎实开展知名民企合作，推动瑞晶电子产业园、中港IC产业园等17个省调度项目尽早竣工。加强与央企合作，力促中建材1.5万吨高强度空心玻璃微珠、国显液晶显示模组二期等落地开工。严格落实“四督四保”制度，建成运行项目网上调度平台，健全推进和责任追究体系，提高项目开工率、竣工率、达产率、转化率。开展项目谋划专项行动，建立五大发展行动重点项目库，主动对接国家重大工程包、专项建设基金等政策，上半年新增项目储备规模3千亿元以上，全年达到1万亿元。 

　　积极培育消费新热点。加快发展线上线下的新业态，加强电商核心竞争力建设，鼓励电商、快递企业通过“互联网+”拓展空间、融合发展。蚌埠电商产业园、蚌山跨境电商产业园争创省级示范园区。加快推进新港物流园、高远物流园、皖北杂粮市场等建设，积极申报国家物流标准化试点城市。深入推进“15分钟”便民服务圈和绿色消费圈建设。引进天天、韵达等快递知名企业建设皖北分拨中心、仓储物流配送中心，逐步实现乡镇有网点、村村通快递。 

　　（四）加快振兴县域经济，开拓更大发展空间 

　　加快县域工业化和新型城镇化进程。落实和完善促进县域经济发展政策，放大三县产业发展比较优势。支持怀远县突出新型城镇化、推动产城融合发展，五河县突出生态立县、推动绿色崛起，固镇县突出现代农业、推动一二三产融合。牢固树立工业强县理念，支持县域特色经济、配套经济发展，推动县域产业融入全市产业链和创新体系，全面推进工业园区基础设施配套建设。支持蚌埠铜陵（固镇）现代产业园加快发展，推动怀远龙亢经开区建设绿色食品基地，加快蚌埠铜陵（五河）现代产业园建设，三县开发区规上工业总产值占全市比重提高1个百分点，二、三产比重提高1.5个百分点。投资20亿元以上工业项目用地实行全市统筹，在产业发展基金等方面给予支持。设立农业产业化基金，着力发展农产品精深加工、主食加工和综合利用加工，新增规上农产品加工企业10家，规上农产品加工业产值达900亿元，主要农产品加工转化率60%以上。深化城镇建设管理提升行动，三县完成大建设投资215亿元。推动中心城市教育、医疗等优质资源下沉，提升县域基本公共服务水平。实施“县道提升、乡道畅通”工程，建设县乡公路270公里、乡村道路530公里，改造30户以上自然村泥水路600公里。开展22个乡镇政府驻地建成区整治，完成26个省级中心村和34个市级、38个县级美丽乡村建设。提升农村人居环境质量，推进农村垃圾清扫保洁全部市场化，实施沟渠疏浚、贯通、补水工程，积极开展村庄房前屋后绿化。 

　　推进农业供给侧结构性改革。坚持“藏粮于地”、“藏粮于技”，深入开展粮食绿色增产模式攻关，粮食产量280万吨。统筹调整粮经饲种植结构，优化糯稻、杂粮等经济作物品种品质和区域布局。创建国家和省级蔬菜标准园6个。新增大水面生态养殖面积2万亩，创建3个部、省级畜禽养殖业示范场，支持华信禽业等龙头企业发展。实施农业品牌提升工程，创建市级以上农产品品牌20个，新增农产品认证30个。启动创建市级农业社会化服务示范乡镇和示范主体。新增农业新型经营主体500家、服务主体100家。主要农作物机械化耕种收综合水平达81%。创建省级农业产业化示范区，产业集群产值达100亿元1个、50亿元3个。三县各引进1个产值10亿元以上的一二三产融合项目。按照“一镇一业、一村一品”要求，大力发展“互联网+”特色城镇经济和乡村旅游经济。着力推进适度面积土地流转。实施新一轮水利设施薄弱环节建设性治理，推进五河整县沟渠连通补水蓄水、淮河干流一般堤防除险加固、西淝河洼地应急治理、天河张公湖龙子湖水系连通等重点水利工程建设。全面完成全域永久基本农田调整工作，全市耕地保有量不低于562万亩。 

　　深化农村综合改革。坚持“政府引导、农民自愿、有序推进”原则，充分利用农村土地确权登记颁证成果，加快实施农村土地所有权、承包权、经营权分置改革。有序推广“一户一块田”。推动农村集体资产股份合作制改革和资源变资产、资金变股金、农民变股东改革，稳步推进农村土地承包经营权和农民住房财产权“两权”抵押贷款试点，赋予农民更多财产权利，增加财产性收入。实施10个重点镇和经济发达镇行政管理体制改革。开展第二批20个“村改居”工作。培育禾泉、沱湖等特色小镇，每个县区创建1个市级特色小镇，全市争创2-3个省级特色小镇。完成第三次全国农业普查。 

　　（五）加强四个能力建设，提升中心城市能级 

　　提升通达力。强化与淮安、信阳等城市的沟通合作，建立常态工作机制，共同推进淮河生态经济带战略在国家层面实施。启动编制淮河流域中心城市、皖北地区中心城市发展规划和交通综合体系建设规划，一体推进交通、水运、生态、产业、公共服务等领域开发建设。加快蚌五高速公路及G206南段一级公路建设，开工建设G329蚌埠至怀远一级公路、蚌固高速公路、解放南路与凤阳连接线、京台高速东海大道出入口、水蚌线外迁、城市云轨试验段等工程。实施淮河航道等级“三升二”。加快G345黄疃窑淮河大桥、五河-固镇-怀远（机场）-蒙城快速通道、高铁蚌埠南站动车运用所等项目前期工作。力争腾湖民用机场开工。谋划南阳-驻马店-蚌埠-盐城、合肥-新沂蚌埠连接线、三门峡-蚌埠-洋口港等铁路。 

　　夯实承载力。实施大建设项目290个，完成投资400亿元。加大城市征迁改造建设力度，全面完成“363”框架沿线和城市主干路两侧棚户区改造。推进中环线北环、西环（淝河路-淮上大道）和西南环（黄山大道-山香路）等建设，全面贯通中环线。开工建设延安路淮河大桥，实施航华路等25条“断头路”贯通工程，加快朝阳路等74条主次干道“白改黑”建设。加快市体育中心、市体育场综合提升改造、龙子湖体育公园等场馆建设，新建18个街头运动场，规划建设城区体育公园、涂山国家级健身步道。建成城市地下管线综合管理信息系统，完善城市排水防涝工程体系。改建供水管网20公里，铺设天然气管道35公里。开展智能立体停车场建设试点。推进无线城市建设，实现市区主要公共区域免费WIFI全覆盖。 

　　增强吸引力。大力推进大众创业、万众创新，按照“三个留住”要求，积极推进农民工返乡创业园、青年创业园、“双创一条街”建设。实施中心城区落户“五个零门槛”购房政策，实行农民户口变动与“三权”脱钩，保障新市民住房需求。保税物流中心业务量突破4亿美元，启动综合保税区筹建工作，争取纳入省自贸区创建。建成二类水运口岸，加快进境粮食指定口岸等申建工作。推进双墩-禹墟国家考古遗址公园和花鼓灯艺术世界非遗项目申报工作，加大宝兴面粉厂、篾匠街、科学文化宫、二七文化宫等“蚌埠记忆”的保护性开发力度。继续建设国家级大禹文化产业示范园区，启动创建龙子湖（古民居）5A级旅游景区，推进大禹文化活动中心前期工作。开放大明文化产业园、古民居博览园部分景区，加快皖北旅游集散中心、白乳泉景区改造提升、花博园二期、新文化馆建设。建成投用文学艺术中心、妇儿活动中心。引导社会资本上山下乡，积极发展休闲观光、采摘体验、农家乐、民宿等乡村旅游，创建2个以上省级休闲农业示范点。 

　　拓展辐射力。抢抓国家“提高三四线城市教育、医疗等公共服务水平”机遇，加强国内外优质教育、医疗资源引进和培育力度，完善集团化、社会化办学办医体制机制，打造区域教育、医疗中心。新建、改扩建幼儿园33所，建成城南九年一贯制学校、滨湖新区新城实验学校等，开工建设北师大附属学校三期、北大培文学校等项目。加快职教园区建设，确保入园学校秋季顺利开学。建成投用安财商学院新校区、安徽科技学院产学研基地。推进与蚌埠医学院共建医科大学。充分发挥安徽财经大学、蚌埠学院等“智库”作用。坚持以“防未病、看小病、治大病”为重点，深化医药卫生体制综合改革，加快建立以医联体、医共体和重点学科为依托的分级诊疗体系。推动城乡居民医保整合，改革支付方式，扩大按病种付费覆盖面。加快蚌医总医院、市一院妇产儿童医院、市二院心血管医院、市三院综合改造等建设，力争完成蚌医一附院、二附院在建项目。加快实施淮上中科生态养老示范园、固镇医养一体、怀远老年康复护养院、民政园等医养结合项目，支持蚌埠五院做大医养结合规模，大力发展以社区为依托的居家养老，积极创建国家医养结合试点市。加快推进中恒城市广场、国购广场等建设，建成运营天洋国际大厦、南翔城市广场一期。净增限上批零住餐企业40户、规上服务业企业20户。支持地方金融机构发展，构建个性化、信息化金融服务体系，加快建设区域金融中心。 

　　（六）深化改革开放，持续释放体制机制活力 

　　攻坚重点领域改革。深化商事制度改革，扩大“证照合一”覆盖面，推行全程电子化登记管理和电子营业执照。继续深化国有企业改革，完善市属企业负责人薪酬制度。推进政府投融资平台转型发展，提升基础设施和公益性项目融资能力。主动对接省事权和支出责任划分改革，调整完善市对区财政体制。强化预算约束，规范市本级财政资金审批程序。完善全口径政府债务管理，严格控制新增债务规模和融资成本。实施国税、地税征管体制改革，落实“营改增”试点政策。推进城市执法体制改革，完成城市管理领域机构综合设置。构建“亲”、“清”新型政商关系，提升企业家信心，让企业家专注创新、安心创业、愉快创造。统筹推进创业就业、社会治理等领域综合改革，有力回应社会期待与诉求，让改革带着温度落地。 

　　加大精准招商力度。密切关注科技前沿和产业转移新动态，实行“点对点”精准招商，推进全产业链板块招商、以商引商，探索“互联网+招商”、“招商外包”等新模式，推动京东方、人民电器、青年新能源汽车等落户蚌埠。强化县区、开发区招商主体责任，每个县区至少引进投资20亿元以上产业类项目2个。开展招商引资环境提升年活动，在法定权限范围内完善招商引资优惠政策。全市实际利用市外亿元以上项目资金900亿元。 

　　扩大对外开放合作。加大赴欧美、日韩、台湾等国外、境外招商力度，实际利用外商直接投资16亿美元以上。大力支持骨干企业“走出去”，加快实施丰原集团巴西、匈牙利玉米深加工、蚌埠玻璃工业设计研究院尼日利亚玻璃生产线、蚌埠国合津巴布韦福利中学等项目。 

　　（七）加强生态文明建设，推动绿色低碳循环发展 

　　强化环境综合治理。“要像保护眼睛一样保护生态环境，像对待生命一样对待生态环境”。强化环境保护 “一岗双责”，建立健全生态环境损害评估和赔偿制度，严格落实领导干部自然资源资产离任审计、损害责任终身追究制度。全面加强环境监管工作，对破坏生态环境的行为，绝不手软，绝不搞下不为例。积极配合中央和省环保督察，扎实推进第二次全国污染源普查前期工作。开展蓝天行动，持续加强工业废气、城市扬尘、机动车尾气、餐饮油烟等综合治理，强力推进建筑砂石生产、储运全覆盖全封闭管理，推动市区商砼企业退市进郊，严格实行秸秆禁烧，PM10平均浓度降到73微克/m3。开展清水行动，全面推行“河长制”，强化“一河三湖”为主的水环境综合整治，完成沱湖中上游拆网还湖，实施城市圈堤外滩地环境综合治理二期工程，全面整治黑臭水体，水质优良率75%以上。建设老城区雨污分流管网二期工程、秦集污水提升泵站及配套管网工程。推进县城饮用水水源地安全保障工程，加强农业面源污染治理。开展净土行动，建立土壤环境质量监测网络，强化土壤污染监管执法，生活垃圾无害化处理率达99%。 

　　补齐园区环保设施短板。解决高新区市第四污水处理厂收水不足、进水浓度偏低问题，推进沫河口精细化工园区一期污水处理厂达到一级A排放标准、二期污水处理厂建成投运，完善怀远工业园区污水管网建设，加快蚌埠铜陵（固镇）现代产业园、怀远龙亢经开区污水集中处理设施建设，完成固镇经开区污水处理厂排水改道工程等。 

　　节约高效利用资源。落实能源和水资源消耗、建设用地等总量和强度双控行动，建立以亩均效益为核心的资源环境正向激励和倒逼机制，能源消耗增速控制在2.75%以内。加快推进五河上海电气生物质电厂建设，支持莱姆佳国家级重点实验室开展秸秆综合利用研究，开工建设武汉光谷蓝焰生物综合利用项目，启动建设以秸秆为原料的现代环保产业示范园区。建成运营生活垃圾焚烧发电厂，新建建筑垃圾处理厂、餐厨废弃物处理中心，提高污泥处理率，建设以垃圾综合处理为基础的生态环保产业园。 

　　提升生态系统功能。加快国家生态文明先行示范区建设，积极构建产权清晰、多元参与、激励约束并重、系统完整的生态文明制度体系。综合保护、合理开发三汊河国家湿地公园和沱湖湿地自然保护区。开展林业增绿增效行动，植树造林2万亩，森林抚育20万亩，建设农田林网示范片10个。启动全国绿化模范城市创建工作，深化绿满珠城行动，加快曹山森林公园、虎山公园、陶山植物园等建设，新增城镇园林绿化344万m2，改造绿地225万m2，新建街头游园19个，建成城市绿道50公里，建成区绿化覆盖率达41%。 

　　（八）织密扎牢民生保障网，提升人民群众获得感 

　　全面完成脱贫攻坚年度任务。始终把脱贫攻坚作为重大政治任务和最大民生工程，坚持精准扶贫、精准脱贫基本方略，扎实推进脱贫攻坚九大专项行动，逐户逐人落实帮扶措施，确保减贫21225人以上。增强集体经济薄弱村“造血能力”，大力发展“龙头企业+合作社+贫困户”生产经营模式。建立贫困人口慢性病门诊医疗救助机制，落实 “三保障一兜底”综合医保政策。完成236人易地扶贫搬迁任务。定期开展脱贫成果“回头看”，实现750个有扶贫任务村市县级督查全覆盖。加快形成防逆机制链条，确保真脱贫、不返贫。 

　　巩固提升民生工程。确保财政资金足额及时到位，全面完成33项民生工程。重点抓好健康脱贫兜底“351”及建档立卡贫困患者慢性病费用补充医疗保障“180”、贫困地区农村义务教育学生营养改善、医疗卫生人才能力提升、技工大省技能培训等6项新增民生工程，提标扩面就业扶持、城乡居民基本医疗保险、公共卫生服务及妇幼健康、计生特扶等5项民生工程，继续实施老旧小区整治等22项民生工程。原农村五保供养及运行维护、孤儿基本生活保障、生活无着落人员社会救助等9项民生工程项目整合归并为3项，项目数减少6项，基本内容未改变。 

　　加大民生投入力度。始终把保障和改善民生作为政府工作的根本取向，坚持新增财力向民生倾斜，公共资源向民生倾斜。积极推进“创业珠城”行动计划，新增城镇就业6万人。开工建设各类保障性安居工程2万套，基本建成1.17万套。完善救急难工作机制，推行社会救助信息化建设“固镇模式”。改革完善计划生育服务管理，提升全覆盖生育保障和全流程健康服务水平。加强农村留守儿童关爱保护工作。适应企业和居民需求，开通3条“微循环”试点公交线路，新增、优化公交线路7条，新增新能源公交车300台。 

　　（九）强化文明城市创建，全面提升文明水平 

　　坚持日巡查、周调度、月评比、季兑现、年总评考查考核机制，深入实施交通秩序、经营环境、市民素质等十项提升行动，决战决胜、全力冲刺全国文明城市。 

　　提升文明交通水平，整治交通秩序。整治乱停乱放、机非混行等交通乱象，开展机动车“礼让斑马线”活动，开展非法载客营运的电动车、燃油三轮车、黑头车、“僵尸车”专项整治。提升管理水平，整治经营秩序。开展以整治出店（出线）经营、占道经营、骑路经营“三种经营”为主的专项整治行动。提升经营环境，整治集贸市场。巩固提升12个示范集贸市场的环境和管理水平，年内再完成12个示范集贸市场的改造提升任务。提升全域文明水平，整治乡村环境。整治乡镇政府所在地脏、乱、差现象， 35%以上村镇达到县级及县级以上文明村镇标准，三县达到省级文明县城水平。提升市民文明素质，整治不文明行为。深化“革除陋习，做文明有礼蚌埠人”系列活动，积极倡导文明用语、文明用餐、文明旅游、文明祭祀、文明上网等文明行为。提升市民生活环境，整治老旧小区、背街小巷。整治86个老旧小区、88个背街小巷。提升城市颜值，整治市容市貌。全面推进地面（水面）、立面、空中综合整治改造，以整治脏、乱为主的地面（水面）环境卫生行动，以整治沿街建筑（构筑）物外形、店招店牌杂乱、色调不协调为主的立面行动，以改造各类管线、取缔乱披乱挂、拆除违章建设、油烟乱排放为主的空中整治行动。提升服务功能，整治完善公共基础设施。整治完善主次干道、公园广场等公共设施缺失损毁、坑洼积水，整治各类管线的地面配套设施，增置一批城市街景游园。提升公益广告宣传水平，整治广告乱象。整治各类户外广告乱象，提升公共场所公益宣传档次。提升精神文明建设水平 ，深化群众性创建活动。培育和践行社会主义核心价值观，深入推进“五大创建”，持续开展“诚信蚌埠·孝善珠城”主题系列活动，完善四级志愿服务网络，倡导网络道德和网络文明。 

　　（十）深入推进平安蚌埠建设，维护社会大局和谐稳定 

　　健全公共安全体系。严格落实重大决策社会稳定风险评估制度和重大不稳定问题清单制度，推进“互联网+社区治理、管理和服务”。加强社会治安和社区服务网格化管理，推进网格员队伍专职化。加快推进“天网”四期工程建设和视频整合应用，完善立体化、信息化社会治安防控体系建设，持续开展“守护平安”系列行动，坚决打击非法集资、电信网络诈骗等各类违法犯罪行为。 

　　强化食品药品安全。深入实施食品药品风险分级监督管理，用最严谨的标准、最严格的监管、最严厉的处罚、最严肃的问责，加快构建可追溯、全流程食品安全监管体系。扎实推进各级食品药品安全城市与农产品质量安全县创建工作。深入推进化肥农药使用量零增长工程，开展农产品质量安全可追溯试点。 

　　筑牢安全生产防线。牢固树立发展决不能以牺牲安全为代价的红线意识，贯彻“安全第一、预防为主、综合治理”的方针，突出抓好危化品、特种设备、道路交通等重点领域安全生产，建立健全安全生产风险隐患排查、研判、预警、防范、处置、问责闭环管理机制。加强监管执法，坚决查处违法违规行为，坚决遏制重特大事故发生，切实保障人民群众生命财产安全。 

　　加强和创新社会治理。落实“七五”普法规划，加强法治教育，扩大法律援助。继续推进阳光信访、责任信访、法治信访。深入开展全国和谐社区建设示范城区、示范城市创建活动。推动民族团结进步事业创新发展，依法加强宗教事务管理。扎实推进重点领域信用建设，基本建成公共信用信息征集共享体系，加大对失信行为的惩处力度。加强新形势下全民国防建设、人民防空和双拥优抚安置工作，实现全省双拥模范城“八连冠”和双拥模范县全覆盖。 

　　四、夙夜在公，全面加强政府自身建设 

　　过去的一年，我们扎实开展“两学一做”学习教育和“讲看齐、见行动”学习讨论，确保各项事业沿着党中央确定的正确方向前进。在全省率先公布实施市级公共服务和行政权力中介服务清单，梳理确认公共服务事项1202项，行政权力中介服务事项保留192项、精简41.1%。积极参与、配合市人大常委会《城镇绿化条例》和《城市管理条例（草案）》起草、修改等工作，办理市人大代表议案11件、建议140件，市政协委员提案442件。全市“三公”经费下降34.5%。公务用车运营维护费用减少39.4%。加强党风廉政建设，强化行政监察和审计监督，营造风清气正、干事创业的良好环境。同时，重审批、轻监管现象依然存在，有法不依、执法不严的情况时有发生，部门间协调、配合机制不健全，工作效率不高，不担当、不作为、等靠要的问题仍然不同程度存在，等等。 

　　新的一年，我们将始终保持对事业的敬仰之心、对人民的敬重之心、对权力的敬畏之心，自觉接受、坚决服从市委领导，着力建设为民、务实、清廉、高效政府。 

　　（一）强化“四个意识”，坚守责任担当。牢固树立“四个意识”特别是核心意识、看齐意识，坚定不移地向以习近平同志为核心的党中央看齐，向党的理论和路线方针政策看齐，向党中央决策部署看齐，始终在思想上政治上行动上同党中央保持高度一致。坚持党的原则第一、党的事业第一、人民利益第一，以临渊履薄的危机感、慢进即退的紧迫感、寝食难安的责任感，全力推进蚌埠新一轮大发展大提升。建立健全正向激励和容错纠错机制，为敢闯敢试者撑腰，为创新创业者鼓劲。每一位政府工作人员特别是各级领导干部，都要不忘初心、继续前进，在勇于担当中干事创业，在苦干实干中为民服务，努力做人民满意的公务员！ 

　　（二）强化法治思维，坚持依法行政。积极贯彻《法治政府建设实施纲要（2015-2020）》，让依法办事成为自觉习惯，真正做到再忙不忘法、再急不违法、再亲不枉法。强化重大决策法定程序的刚性约束，所有报送市政府的重大请示事项全部由市法制办进行合法性审查，实现“四维”全覆盖。坚持公开是常态、不公开是例外，强化政务公开标准化建设。认真执行市人大及其常委会的决议和决定，自觉接受人大法律监督、政协民主监督，虚心听取各民主党派、工商联、无党派人士意见，充分发挥工会、共青团、妇联等人民团体桥梁纽带作用，广泛接受社会和舆论监督，把权力关进制度的笼子，让权力在阳光下运行！ 

　　（三）强化职能转变，提高工作效率。坚持权力清单、责任清单动态调整，公共服务清单、行政权力中介服务清单全部进入网上办事大厅办理，全面落实“双随机、一公开”监管机制。牢固树立“今天再晚也是早、明天再早也是晚”的理念，协调解决问题“小事不过夜，大事不过周”。健全“问题导向、举一反三，建立清单、对账销号，完善制度、长效管理”的工作机制，严格落实重点工作和项目“周分析调度、月督查推进”制度。倡导快节奏、高效率的“快乐工作法”，开展办公桌每天“清零”行动，文件签批日清日结，常务会议纪要、专题会议纪要会后1天内签发，审议通过的文件会后3天内印发。实行政府年度会议计划管理，落实“无会周”制度，把“无会周”变成“招商周”、“项目周”。实现政府及组成部门机关协同办公系统一体化，建成运行掌上移动办公系统，用便捷高效的工作平台服务人民、推动发展！ 

　　（四）强化民本理念，加快转变作风。牢固树立以人民为中心的发展思想，多谋民生之利、多办民生之事、多解民生之忧，把执政为民理念贯穿到政府工作的各领域、全过程。深入开展“三访三促”活动，通过访基层企业、访基层组织、访基层群众，促进政府科学决策、政策落实和公信力提升。推进“互联网+政务服务”，实现市县区大数据共享，全面推进网上审批。开展“减证便民”专项行动，切实加强基层为民服务体系建设，打通联系服务群众“最后一公里”。健全网民诉求和网络舆情办理处置机制，相关责任单位在第一时间答复网民诉求。加强政风和机关效能建设，坚决杜绝光说不练胡乱哈、推诿扯皮踢皮球、虎头蛇尾炒剩饭、年初不努力年底抓瞎的不良风气，坚决杜绝不调查研究、不结合实际、简单转发文件的“假落实、乱落实”。坚持抓重点、重点抓，在全面做好各项工作的同时，着力调度推进四个十项重点，即实施十大创新工程、建成十大工业项目、落实十大富民举措、推进十大文明提升，让居民收入、企业利润、财政收入“三个口袋”更加充实，不断提高群众获得感和城市竞争力！ 

　　（五）强化廉政建设，严守纪律规矩。认真贯彻执行《准则》、《条例》，严格落实全面从严治党主体责任，提升政府系统从严管党治党水平。切实履行“一岗双责”，全面加强反腐倡廉制度建设，强化对人权、事权、财权的刚性约束。加强行政监察和审计监督，对公共资金、国有资产、国有资源等重点领域从严监管。编印机关干部正负双面言行“激励+提醒”读本，严格规范政府工作人员言行。牢固树立过紧日子的思想，厉行勤俭节约，反对铺张浪费，把有限的财力用到发展最重要、群众最需要的地方。严肃查处以权谋私、失职渎职案件和腐败问题，切实做到干部清正、政府清廉、政治清明！ 

　　各位代表！ 

　　重任在肩，唯有夙夜在公、撸起袖子加油干，才能不负重托。让我们紧密团结在以习近平同志为核心的党中央周围，在省委、省政府和市委的坚强领导下，凝心聚力、开拓进取，一心一意谋发展，聚精会神抓党建，全心全意为人民，为建成淮河流域和皖北地区中心城市、全面建成小康社会、全面迈入全省第一方阵而努力奋斗，以优异成绩迎接党的十九大胜利召开！
